# Changes in Achilles tendon stiffness and energy cost following a prolonged run in trained distance runners

**DOI:** 10.1371/journal.pone.0202026

**Published:** 2018-08-08

**Authors:** Jared R. Fletcher, Brian R. MacIntosh

**Affiliations:** 1 Human Performance Laboratory, Faculty of Kinesiology, University of Calgary, Calgary, Canada; 2 Department of Health and Physical Education, Mount Royal University, Calgary, Canada; Queen Mary University of London, UNITED KINGDOM

## Abstract

During prolonged running, the magnitude of Achilles tendon (AT) length change may increase, resulting in increased tendon strain energy return with each step. AT elongation might also affect the magnitude of triceps surae (TS) muscle shortening and shortening velocity, requiring greater activation and increased muscle energy cost. Therefore, we aimed to quantify the tendon strain energy return and muscle energy cost necessary to allow energy storage to occur prior to and following prolonged running. 14 trained male (n = 10) and female (n = 4) distance runners (24±4 years, 1.72±0.09 m, 61±10 kg, $\dot{V}O_{2}\text{max}$ 64.6±5.8 ml•kg^-1^•min^-1^) ran 90 minutes (RUN) at approximately 85% of lactate threshold speed (sLT). Prior to and following RUN, AT stiffness and running energy cost (E_run_) at 85% sLT were determined. AT energy return was calculated from AT stiffness, measured with dynamometry and ultrasound and estimated TS force during stance. TS energy cost was estimated on the basis of AT force and assumed crossbridge mechanics and energetics. Following RUN, AT stiffness was reduced from 328±172 N•mm^-1^ to 299±148 N•mm^-1^ (p = 0.022). E_run_ increased from 4.56±0.32 J•kg^-1^•m^-1^ to 4.62±0.32 J•kg^-1^•m^-1^ (p = 0.049). Estimated AT energy return was not different following RUN (p = 0.99). Estimated TS muscle energy cost increased significantly by 11.8±12.3 J•stride^-1^, (p = 0.0034), accounting for much of the post-RUN increase in E_run_ (8.6±14.5 J•stride^-1^,r^2^ = 0.31). These results demonstrate that a prolonged, submaximal run can reduce AT stiffness and increase E_run_ in trained runners, and that the elevated TS energy cost contributes substantially to the elevated E_run_.

## Introduction

The energy cost of running (E_run_) is one of the key determinants of distance running performance [1]. E_run_ is primarily determined by the energy cost of generating the force needed to support body weight for the duration of the stride (2 footstrikes per stride) [2–4] and there is growing evidence to suggest that mechanical properties of the tendons of the major force-generating muscles that are active during running greatly influence E_run_ [5–7]. It has been suggested that the mechanical properties of tendon allow a ‘decoupling’ between the length change of the muscle fascicles to that of the whole muscle-tendon unit, allowing the muscle fascicles to shorten at a much slower speed while high-velocity shortening can be achieved by the recoil of the tendon [8,9]. This minimized shortening magnitude and shortening velocity will minimize the required level of muscle activation needed to generate the necessary force, due to the muscle’s force-velocity relationship [10,11].

The Achilles tendon (AT) undergoes rapid lengthening and shortening during the stance phase of running, taking up much of the length change of the whole muscle-tendon unit, and reducing the need for muscle fibre lengthening and shortening [8,9,11]. The muscle energy cost should be lowest when the AT is mechanically ‘tuned’ to allow minimal muscle fascicle shortening during the stance phase and much of the length change of the muscle-tendon unit can be accommodated by the AT.

When cyclically loaded, during running of sufficient duration, the AT may develop dynamic creep [12], resulting in greater length change for a given load. Dynamic creep can be assessed by measuring changes in AT stiffness following a run. If significant dynamic creep does occur during/following a prolonged run, this implies that for any muscle-tendon unit length and the same force requirements during stance, AT elongation would be greater, necessitating greater TS fascicle shortening. This additional fascicle shortening would require more muscle energy [10,13], which would be proportional to the additional shortening, due to shortening-induced increases in ATP turnover [14] and higher shortening velocity [15,16]. The additional muscle energy cost should contribute to an elevated E_run_ although the measurement of E_run_ and/or fascicle shortening during running may lack sufficient sensitivity to demonstrate a meaningful causation. Indeed, Albracht and Arampatzis [17] have previously demonstrated that E_run_ was significantly reduced when AT stiffness was elevated following a period of resistance training but could not show a significant difference in MG fascicle shortening. No studies to date have evaluated the opposite effect. That is, does a reduction in AT stiffness result in concomitant increases in E_run_?

Bouts of long distance running of 60–90 minutes have been shown to acutely elevate E_run_ in moderately-trained runners [18–20]. Repetitive contractions, have been shown to decrease AT stiffness [21,22]; however, a recent review [23] suggests prolonged running has only a trivial effect on altering tendon material properties. Specifically, Park et al. (2011) showed no difference in AT stiffness following a short warm-up consisting of a 6-min jog at 180 m·min^-1^ and a series of static stretches. Similarly, Farris et al. [24] could not demonstrate a significant reduction in AT stiffness following a 30 min run at 200 m·min^-1^ and only in older runners did AT stiffness decrease following a “half marathon” run (20.5 km) at 186 m·min^-1^. AT stiffness was unaltered by a 20.5 km run at 216 m·min^-1^ in young runners [25], confirming the results of Lichtwark et al. [26] following a 5 km run. However, the latter authors showed a small but significant increase in tendon strain following the run. Increased tendon strain would allow greater energy storage, but also likely results in an elevated muscle energy cost [27]. The interaction between elevated tendon strain energy storage/return and muscle energy cost has to date not been investigated theoretically. Thus, the purpose of this study was to investigate the impact of a prolonged, submaximal run of 90 minutes on potential changes in AT stiffness, estimated strain energy return and muscle energy cost. We set out to test the null hypothesis that there would be no change in AT stiffness, strain energy return, E_run_ or muscle energy cost following a prolonged, submaximal run.

## Materials and methods

14 trained male (n = 10) and female (n = 4) distance runners participated in this study. It was anticipated that the male and female runners would demonstrate a range of values for E_run_ and AT stiffness. Subject characteristics are shown in Table 1. All runners were training regularly at least 6 times per week and following a similar, periodized training plan designed for peak 5000m or 10000m performance for the females and males, respectively. Average weekly training volume was 80±7 km•wk^-1^ and 101±28 km•wk^-1^ for the female and male runners, respectively. None of the runners had any neuromuscular or musculoskeletal injuries at the time of the study. The runners gave their informed written consent for the experimental procedures., The University of Calgary Conjoint Health Research Ethics Board reviewed and approved this study.

**Table 1:** Subject characteristics.

	N	Age (years)	Height (m)	Mass (kg)	$\dot{V}O2max$ (ml·kg^-1^·min^-1^)	sLT (m·min^-1^)
Male	10	25.8±4.5	1.76±0.06	66.3±6.7	66.8±4.7	265.4±15.2
Female	4	21.0±0.8	1.61±0.05	47.8±2.6	59.0±4.3	234.8±18.6
Total	12	24.4±4.4	1.72±0.09	61.0±10.4	64.6±5.7	256.7±21.1

Values are mean±sd.

**Fig 1:**
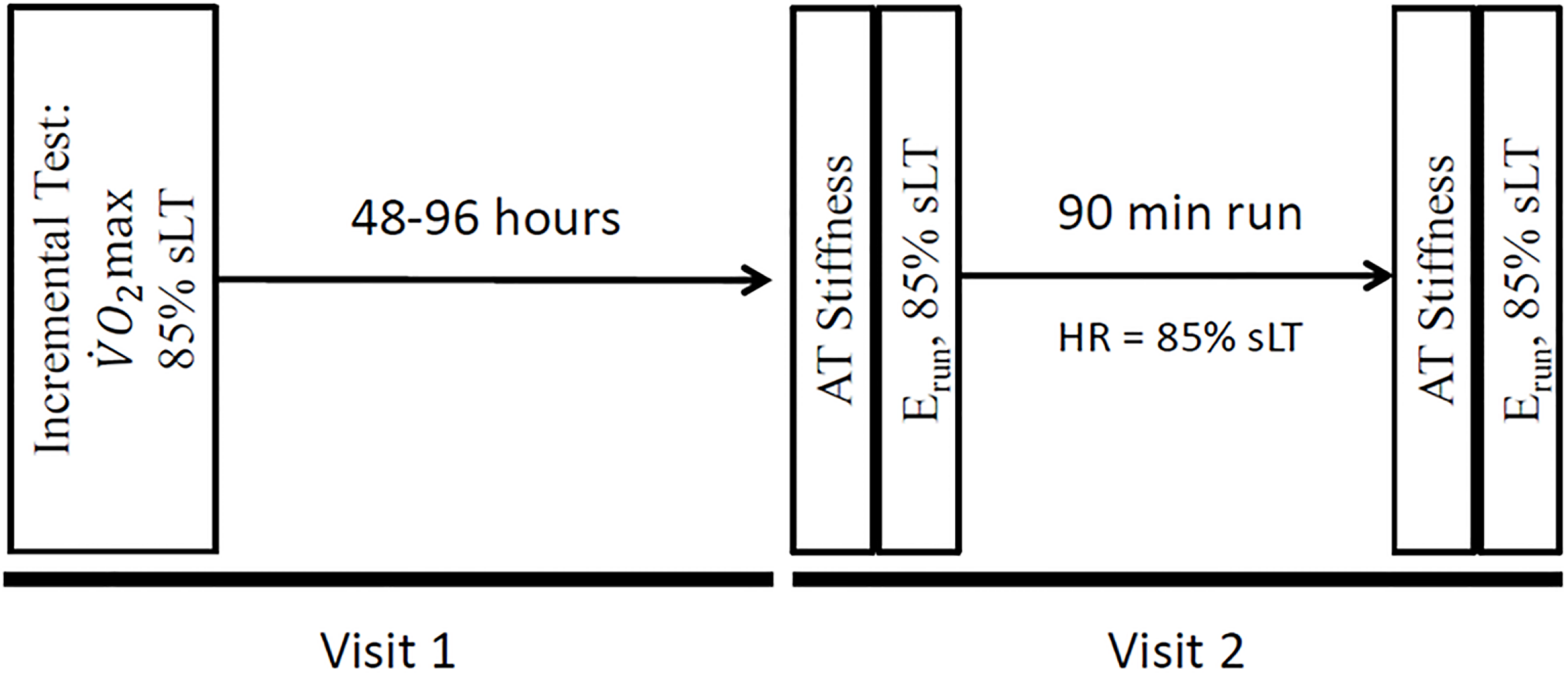
Experimental protocol. On the first visit to the lab (*Visit 1*), the subject’s VO_2max_ and 85% sLT were determined. The subjects returned to the lab 48–96 hours later (*Visit 2*), when AT stiffness and E_run_ at 85% sLT were determined before and after a 90 min run at a prescribed heart rate associated with 85% sLT.

The subjects visited the lab on two separate occasions. The experimental protocol is shown in Fig 1. During the first visit, an incremental test to exhaustion was performed on a treadmill (Woodway Pro, Woodway USA, Waukeshka, WA) to determine the subject’s maximal oxygen uptake ($\dot{\text{V}}{\text{O}}_{2}\text{max}$) and speed associated with the lactate threshold (sLT). The methods used to determine $\dot{\text{V}}{\text{O}}_{2}\text{max}$ and sLT were the same as those methods used previously in our lab and described elsewhere [28–30]. Briefly, subjects began running at approximately 3 km·hr^-1^ below their most recent 10 km race performance for 2 min. The speed was increased by 0.8 km·hr^-1^ every two minutes. At the end of each 2 min stage, a fingertip blood sample was taken for lactate determination (Lactate Pro). Once blood lactate concentration rose more than 1 mM from the previous sample, the treadmill speed was decreased by 0.8 km·hr^-1^ and the grade was increased 2% every minute until volitional exhaustion. This speed was considered the sLT. $\dot{\text{V}}{\text{O}}_{2}\text{max}$ was considered the highest 30s average $\dot{\text{V}}{\text{O}}_{2}$ during the incremental test. All incremental tests were terminated due to volitional exhaustion and all subjects attained $\dot{\text{V}}{\text{O}}_{2}\text{max}$ based on primary or secondary criteria [28].

Between 48 and 96 hours following the first laboratory visit, the subjects returned to the lab for determination of AT stiffness and E_run_. Subjects then completed a prolonged, 90 minute run (RUN). RUN was performed over level ground on an outdoor road loop surface (in the subject’s own running shoes) at a pace corresponding to approximately 85% of sLT. Care was taken to ensure similar running environmental conditions between subjects (14–19° C, 38–54% relative humidity). The speed and heart rate associated with 85% sLT were determined from the incremental test to ensure consistent relative pacing between subjects. Each subject wore a heart rate monitor (Suunto t6c, Oy, Finland) during RUN and was instructed to maintain the heart rate corresponding to 85% sLT throughout. Since these were experienced runners, they were also given information prior to RUN regarding the approximate target speed (minutes•km^-1^ and minutes•mile^-1^). Following RUN, heart rate data were downloaded to a computer using the manufacturer’s software (Suunto Team Manager, Suunto, Oy, Finland). The average (±2 SD) heart rate was calculated and only those runs for which the average heart rate was within ± 3 beats•min^-1^ of the target heart rate through-out RUN were used for subsequent analysis for AT stiffness and E_run_. In total, five RUN trials (4 men and 1 woman) had to be repeated 7 days later based on the above exclusion criterion. Average heart rate during RUN was used to estimate approximate run speed, from the heart rate-speed (below sLT) relationship determined during the incremental test.

### Measurement of E_run_

Immediately following the measurement of AT stiffness and before RUN, a 5-minute warm up at 133 m•min^-1^ for the females and 160 m•min^-1^ for the males was performed on a motorized treadmill (Woodway Pro, Woodway USA, Waukesha, WI). The measurement of E_run_ was performed following the warm-up by having the subjects run in the same shoes worn during RUN at 85% sLT for five minutes. Following RUN and the measurement of post-RUN AT stiffness, E_run_ was measured on the same treadmill at the same relative speed. No cool-down was performed following RUN. $\dot{V}O_{2}$ was measured throughout the 5-minute run using a metabolic measurement cart (TrueMax 2400, Parvomedics, Salt Lake City, UT). The cart was calibrated before and after each session with a two-point calibration using room air and a gas mixture of known composition (4% CO_2_, 16% O_2_) and a manual 3-L syringe. According to the manufacturer, the accuracy of this system is 0.03% and 0.1% for O_2_ and CO_2_, respectively and ±2% for volume.

E_run_ was calculated from the steady-state $\dot{V}O_{2}$ and respiratory exchange ratio (RER) over the final 2 minutes of the 5-minute stage. E_run_ was expressed in units of energy (J•kg^-1^•m^-1^), as described previously [30,31].

### Determination of AT stiffness

Prior to and immediately following RUN (ie. within 2 minutes), AT stiffness was determined on the subject’s right leg using methods described previously [27–29] and detailed here. Three isometric ramp maximal voluntary contractions (MVC) of the right plantarflexors were performed on a dynamometer (Biodex System 3, Biodex Medical Systems Inc., Shirley, NY, USA). The shank and unshod foot were affixed to the dynamometer using a series of Velcro straps The highest measured MVC was used for subsequent analysis. The first MVC was performed less than 2 minutes following the completion of RUN in all subjects. During the MVC, a 12.5 MHz linear array ultrasound probe (50mm, Philips Envisor, Philips Healthcare, Eindhoven, Netherlands) was used to visualize the medial gastrocnemius muscle (MG) fascicles at a frequency of 49 Hz. Contrary to the methods of Fletcher et al. [29] who measured tendon translation clearly on the muscle belly, we chose to measure tendon translation near the myotendinous junction. This was done to reduce any effect of aponeurosis compliance on the measurement of tendon elongation [32] while still being able to visualize a series of MG fascicles. The probe was secured using a custom-built apparatus and elastic straps. AT tendon elongation was estimated by the displacement of an insertion of a fascicle into the deep aponeurosis as close as possible to the myotendinous junction, measured during the MVC, using ImageJ, (NIH, Baltimore, MD, USA). Measured moments and AT elongations were synchronized using an external function generator (B-K Precision 3010, Dynascan Corp, Chicago, IL USA), which was manually initiated at the start of each contraction. Ankle joint rotation was detected by video analysis. The apparent tendon translation due to joint rotation was corrected, knowing the passive joint angle-tendon translation relationship [10,29]. AT moment arm length at the 90° ankle angle was estimated using the tendon travel method [33] by passively rotating the subject’s ankle joint from maximal plantarflexion to maximal dorsiflexion at 0.17 rad·s^-1^ and measuring tendon displacement, corrected for passive force using ultrasound [34]. AT force was calculated by dividing the ankle joint moment by the estimated AT moment arm. AT Force (F)-elongation (d_L_) data were fitted to a quadratic regression equation using:

$$F=Ad_{L}^{2}+Bd_{L}$$ (1)

where A and B are constants. In order to account for any difference in MVC force prior to and following RUN, AT stiffness was defined as the slope of the fitted F-d_L_ equation from 50–100% of maximum isometric plantarflexion force prior to RUN.

### AT energy storage/release and muscle energy cost

AT energy storage and release, as well as muscle energy cost to allow AT energy storage to occur were estimated according to Fletcher and MacIntosh [27]. Briefly, AT energy storage was calculated as the area under the F-d_L_ curve, where F was estimated from the assumed average peak vertical ground reaction forces and running speed [35] and assuming the moment arm of F_z_ during the stance phase was 1.5x greater than the AT moment arm [36]. We acknowledge that this length is not fixed during the stance phase and is different between rear and midfoot strikers [37]. However, we chose a fixed F_z_:AT moment arm ratio since in some cases, a ratio larger than this resulted in an estimated AT force during running which was greater than the maximum isometric force. It seems unlikely that the AT force during running at the prescribed speed would be near the maximum isometric force. This over-estimation of the AT force would result in both an over-estimation of the AT energy release as well as of the estimated muscle energy cost. We further assumed that F_z_ was similar prior to and following RUN [38–40]. A reduction in F_z_ following run (for example, with an increase in stride frequency), would result in a reduced AT energy release as well as a reduced muscle energy cost.

The corresponding tendon length change (d_L_) during running was estimated from each subject’s measured F-d_L_ curve (see Eq 1) and expected joint rotation, a conservative but reasonable estimate of ankle joint rotation at the time of peak vertical ground reaction force [9,38]. The shortening due to joint rotation was added to the shortening due to force-dependent stretch of the tendon to calculate total shortening during the stance phase. AT energy storage was calculated and AT energy release, was estimated assuming an AT hysteresis of 10% [41].

TS muscle energy cost for AT storage/release to occur was calculated from the estimated AT force and fascicle shortening during two sequential footstrikes (one stride), described elsewhere [27]. Briefly, TS force was estimated by calculating the force needed in this muscle during two stance phases (one stride). The number of active crossbridges needed to generate the required force during stance was estimated, assuming a crossbridge force of 3 pN per crossbridge [42]. The number of active crossbridges was then multiplied by the number of crossbridge cycles, based on the amount of shortening expected in each sarcomere, the difference between resting sarcomere length and the sarcomere length during stance. Both sarcomere lengths were extrapolated from the estimated fascicle length-sarcomere length relationship. We assumed this sarcomere length to be 2.6 μm at MVC which is the sarcomere length expected for human muscle at the short end of the plateau of the force-length relationship [43]. A linear increase in force as a function of fascicle length, with no evidence of a plateau [44] suggests that during maximal contractions, the MG operates on the ascending limb of the force-length relationship. Thus, the longest sarcomere length could be no longer than the short end of the plateau of the force-length relationship. The number of sarcomeres in series was estimated by dividing the assumed sarcomere length into the known fascicle length. We further assumed each sarcomere shortened by 10 nm with each crossbridge cycle [45] and that 48 kJ of energy were used per crossbridge cycle [46]. For greater detail, see Fletcher and MacIntosh [27,47].

### Statistics

Values are presented as mean ± SD unless otherwise indicated. Two-tailed paired t-tests were used to test for differences between pre and post-run values for E_run_, MVC force, AT stiffness, AT energy release and muscle energy cost. One-way ANOVA was used to test for differences in d_L_ across absolute force levels. Linear regression analysis was used to examine the relationship between AT stiffness and E_run_, prior to and following the run as well as to examine the relationships between the change in AT stiffness, and the change in E_run_, and the change in AT energy release and muscle energy cost following RUN. All analyses were performed using GraphPad Prism version 6.04 for Windows (GraphPad Software, La Jolla, CA, USA, www.graphpad.com). The *a priori* level of statistical significance was set at *P*<0.05.

## Results

Mean heart rate during RUN was 139±9 b•min^-1^, equivalent to the heart rate at 83.6±4.1% sLT. This corresponds to an approximate run speed of 214.1±13.7 m•min^-1^. This speed was not different than the anticipated speed associated with 85% sLT (218.2±17.9 m•min^-1^, p = 0.175). The average run distance was 19.3±1.2 km.

**Fig 2:**
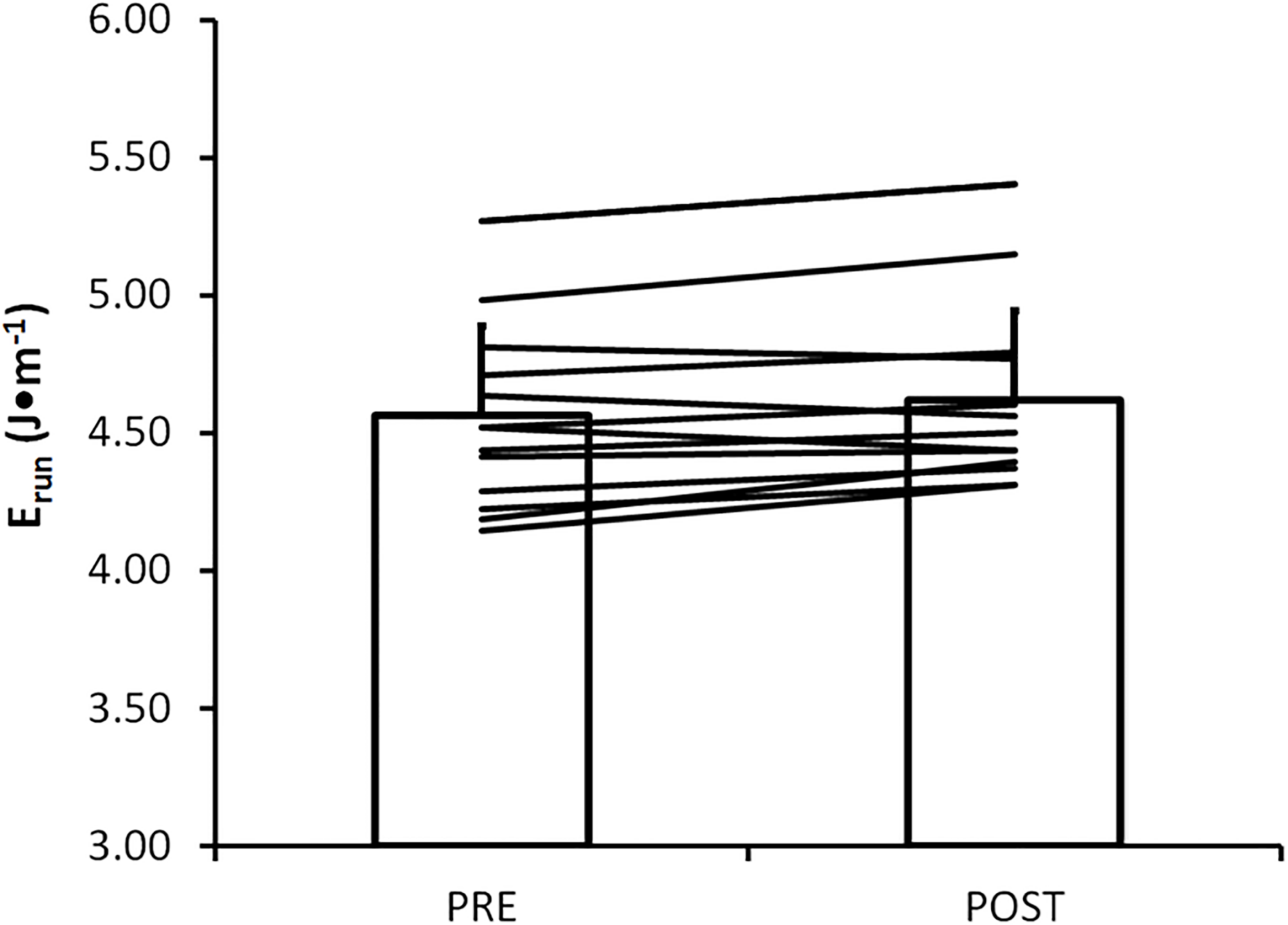
E_run_ prior to (PRE) and following (POST) 90 min run. Black lines represent individual subject responses. E_run_ was significantly elevated (p = 0.044) following 90 min run.

E_run_ prior to and following RUN is shown in Fig 2. Following RUN, E_run_ was significantly higher (p = 0.049) compared to E_run_ measured prior to RUN. This represents a mean increase in E_run_ of 0.06±0.10 J•kg^-1•^m^-1^ (1.3%) following RUN.

**Fig 3:**
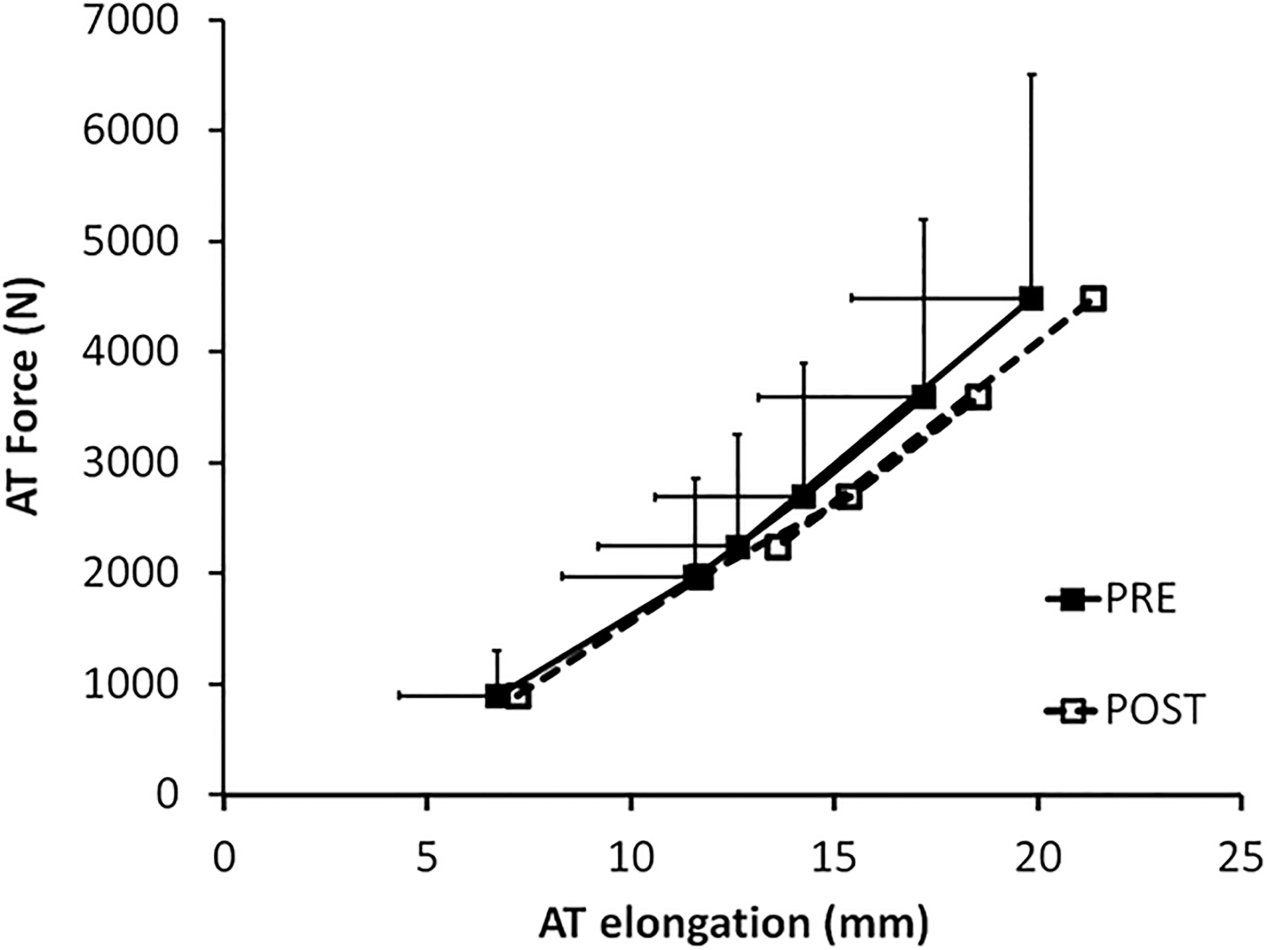
AT force-elongation curve prior to and following 90 min run. **d_L_** is shown at the same absolute force level (shown at 20% MVC-PRE force increments). At all absolute forces, assessed, d_L_ was significantly higher POST-RUN (p<0.001).

**Fig 4:**
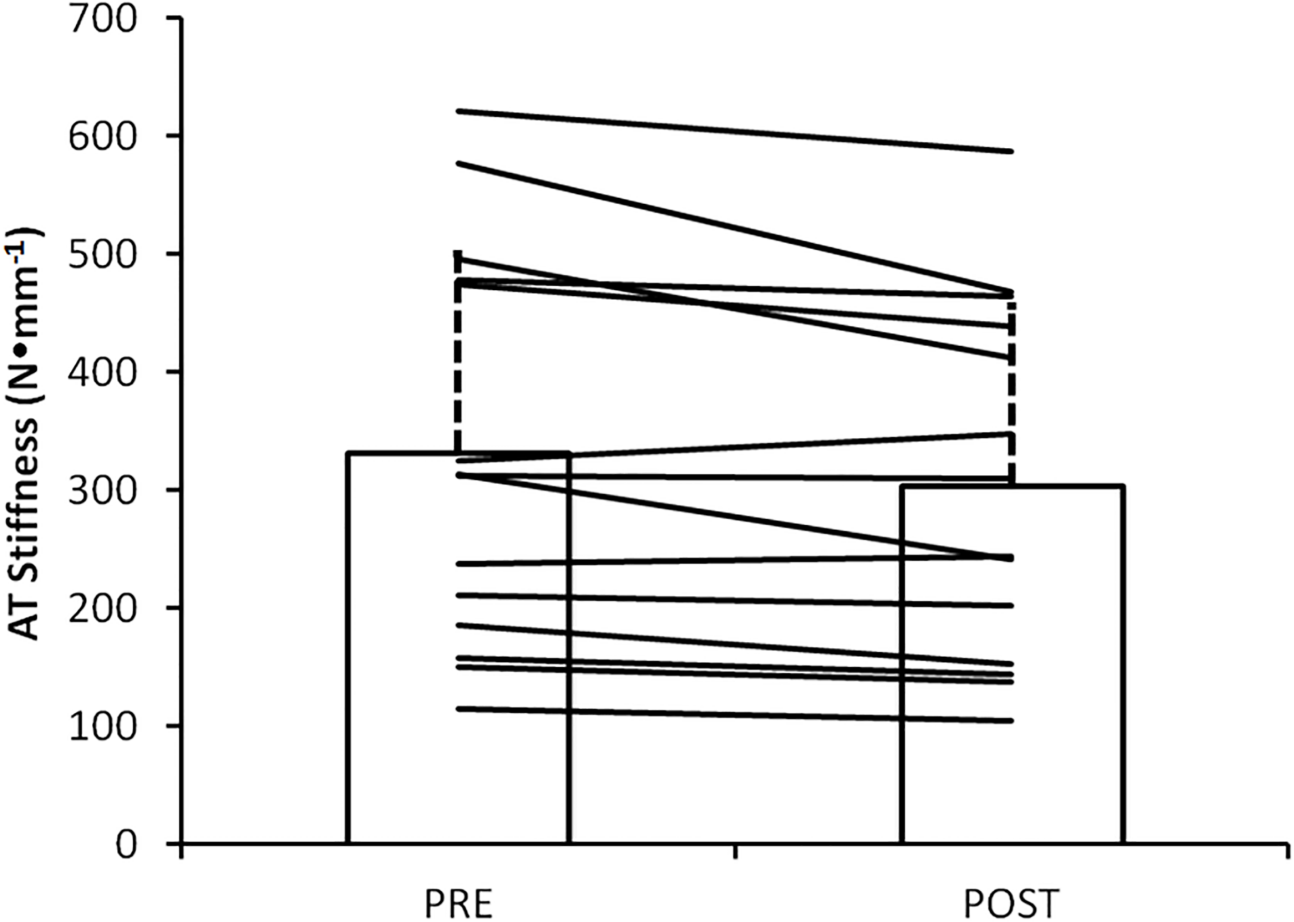
AT stiffness measured prior to (PRE) and following (POST) 90 min run. Solid lines represent individual subject responses. AT stiffness was significantly lower (p = 0.009) following 90 min run.

MVC force was reduced by 3.0±5.7% following RUN, from 4489±2013 N to 4333±1917 N. The 95% C.I. for the difference (POST-PRE) was -312 to 1 N. This difference was not significant (p = 0.0512). The F-d_L_ relationship prior to and following RUN is shown in Fig 3. AT stiffness prior to and following RUN are shown in Fig 4. AT stiffness was reduced following RUN by 28.5±36.5 N•mm^-1^. This reduction in AT stiffness was significant (p = 0.009).

**Fig 5:**
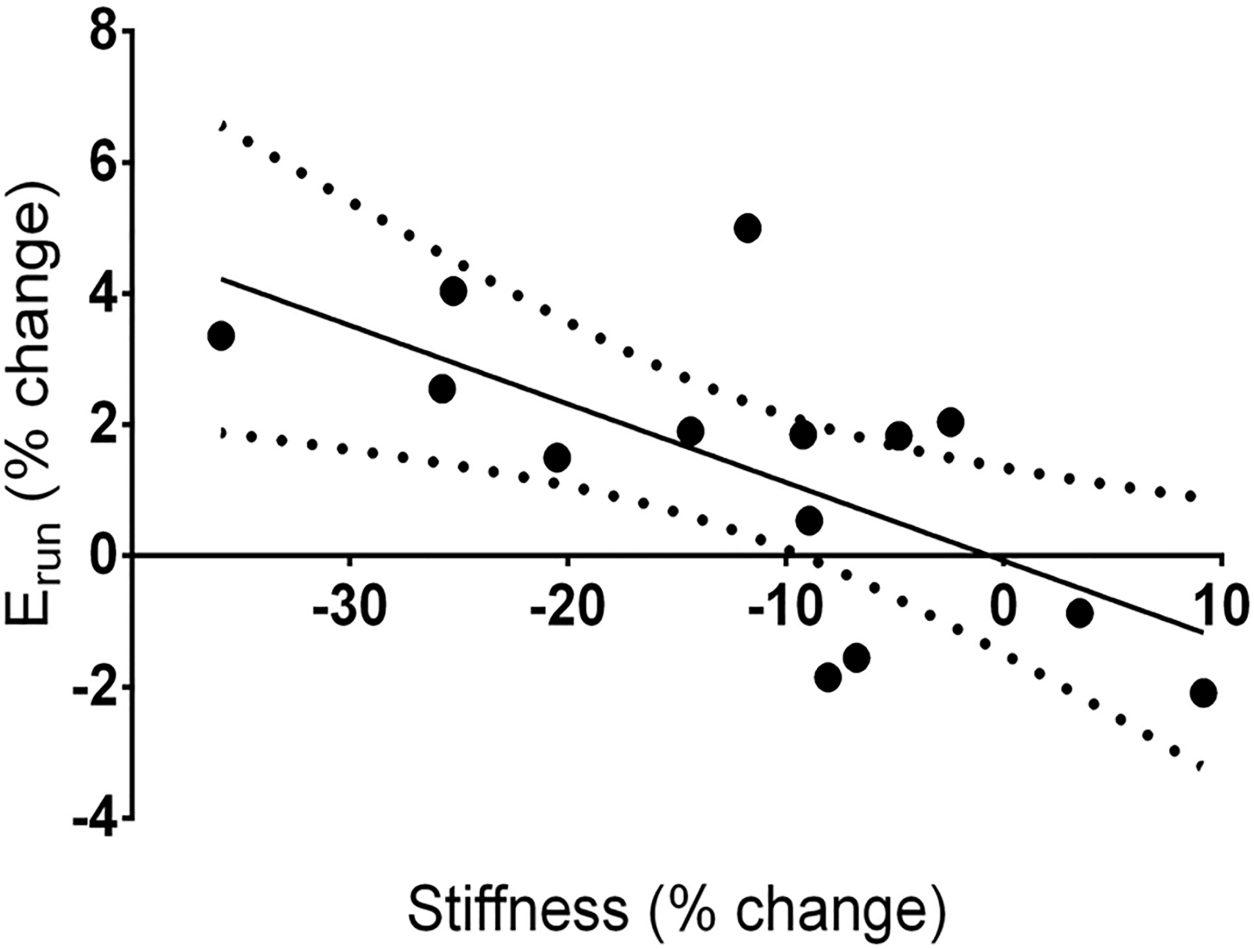
Relationship between the change in E_run_ and AT stiffness following 90 min run. The solid line represents the linear relationship between E_run_ and stiffness (r^2^ = 0.430, p = 0.011). Dashed lines represent the 95% confidence interval for the relationship. Note that the relationship crosses the abscissa at 0% change in stiffness.

Considering the measurements prior to and after the run, there was a significant relationship (r^2^ = 0.430, p = 0.011) between change in E_run_ and change in AT stiffness. A decrease in stiffness was associated with an increase in E_run_ (Fig 5).

Any change in d_L_ and/or force during stance due to RUN would result in a change in the amount of AT energy storage/release. Prior to RUN, we estimated this amount of strain energy release to be 21.6±9.0 J•stride^-1^. Following RUN, AT energy release (21.6±7.5 J•stride^-1^) was not significantly different from PRE (p = 0.996).

**Fig 6:**
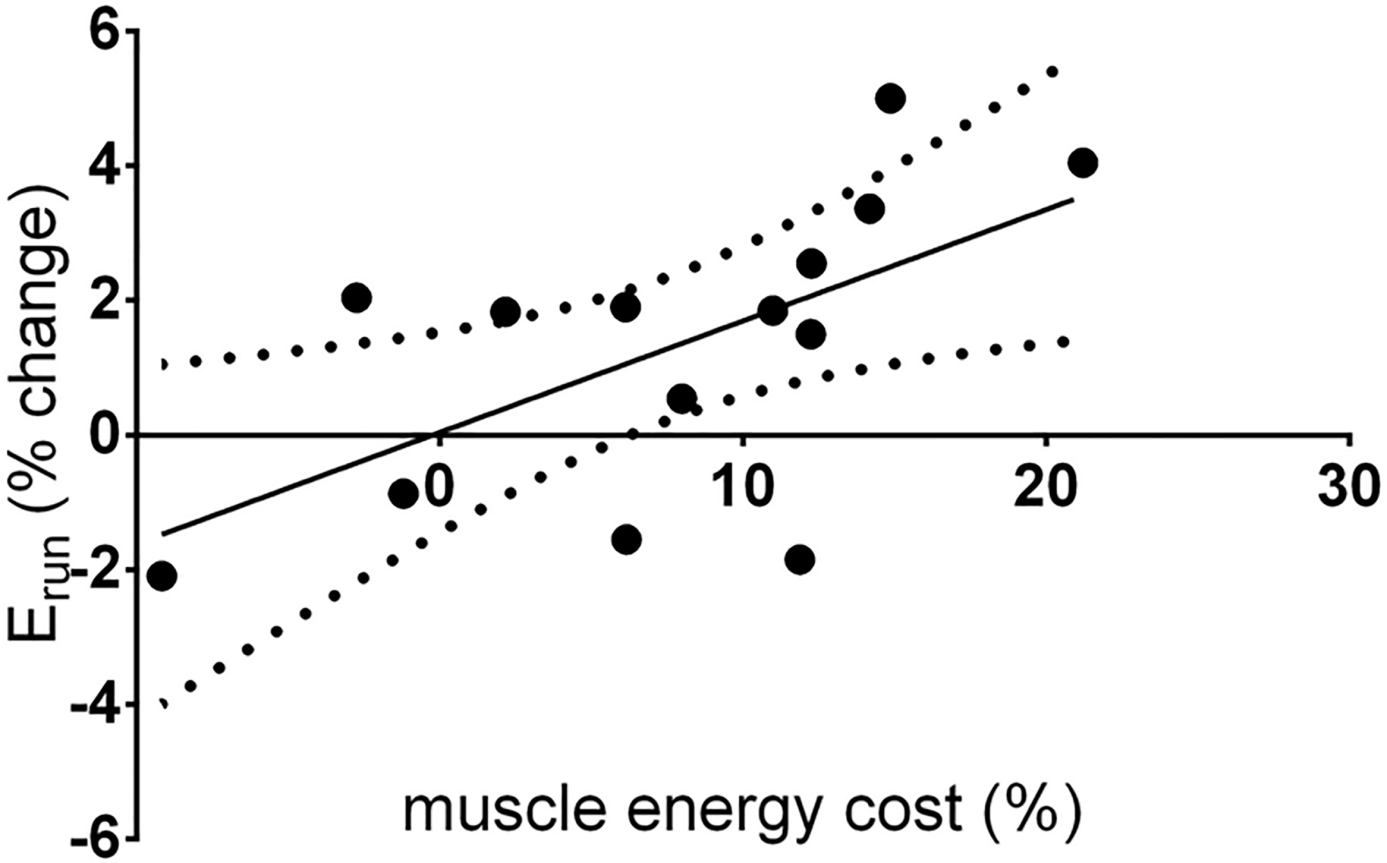
Relationship between the change in E_run_ and TS muscle energy cost following 90 min run. The solid line represents the linear relationship between E_run_ and muscle energy cost (r^2^ = 0.368, p = 0.0213). Dashed lines represent the 95% confidence interval for the relationship. Note that the relationship crosses the abscissa at 0% change in muscle energy cost.

Estimated TS muscle energy cost was significantly elevated following RUN, from 164±61 J•stride^-1^ to 175±62 J•stride^-1^ (p = 0.0034). The change in estimated TS muscle energy cost was significantly related to the change in E_run_ following RUN (r^2^ = 0.368, p = 0.023), suggesting that nearly 40% of the variance in E_run_ can be accounted for by changes in muscle energy cost following RUN. This relationship is shown in Fig 6.

## Discussion

The results of the current study demonstrate that a prolonged, submaximal run similar to that regularly performed in training by distance runners can elicit a small but significant increase in E_run_ and a reduction in AT stiffness. The changes in E_run_ and AT stiffness as a result of this RUN were significantly related, confirming previous reports that a change in AT stiffness is associated with a change in E_run_ in highly-trained distance runners [17,29]. Our estimates of TS muscle energy cost suggest the change in AT stiffness would result in an increase in muscle energy cost, and this might contribute to the measured increase in E_run_. However, we cannot discount that potential post-RUN changes in running kinetics would alter this relationship. We also recognize that our data indicate that some runners apparently decreased their E_run_ after RUN. This possibility requires further research. Further testing to confirm that any changes in running kinetics did not alter muscle forces and/or length change during the post-RUN period are also required.

Changes in E_run_ following prolonged running have been demonstrated in highly-trained runners previously [48]. Specifically, it is reported that runners had a 5.2% increase in E_run_ following a marathon run at 273 m•min^-1^ [18]. Here, we observed a smaller change in E_run_ (1.3%) following a 90 min run at a slower speed (214 m•min^-1^). It seems logical to consider that the change in E_run_ should be dependent on speed and distance run. Brueckner et al. [18] have previously estimated that E_run_ increases as a function of distance run, by approximately 0.08%•km^-1^. Here, we show a similar change of 0.07±0.12%•km^-1^.

It cannot be overlooked, however, that the observed change in E_run_ is small. The magnitude of difference between pre and post-RUN values for E_run_ (1.3%) is smaller than the typical error in measurement of E_run_ of highly-trained runners reported in our lab [30] and others [49]. The smallest worthwhile change (SWC) in E_run_ in highly-trained runners has been reported to be 2.7% when E_run_ is expressed in terms of energy [49]. However, data from our laboratory for trained and elite male and female distance runners suggest the SWC in E_run_ is between 0.8% and 1.1% [27,30]; this SWC is smaller than the magnitude of change we report in E_run_ following RUN. Although we must be cautious in the interpretation that the changes seen post-RUN are ‘real’ and ‘worthwhile’ and not simply related to testing error and typical variation of E_run_, the fact that the difference did reach significance, probably because of the consistency of measurement, is evidence that it is real. Certainly, a change of 1% can impact performance, and many of our subjects showed increases in E_run_ greater than this.

It is interesting to consider the context of a progressive increase in E_run_ and its impact on performance of a record-breaking marathon performance. There was a recent Viewpoint [50] that presented the question: “Who and When” with reference to breaking that record. In the associated Commentaries [51], there was a general consensus that current physiological measures of elite runners are consistent with performance of a 2 hour marathon. However, previous studies and this recent Commentary have ignored the potential that E_run_ may increase progressively throughout the run. This progressive increase in E_run_ has only recently been fully acknowledged [52]. If there is a progressive increase in E_run_ in these runners, then clearly a slow-down would be required if a constant energy cost was necessary for elite performance. A similar change in E_run_ of 0.07% per km over the course of a world-class marathon (eg. 2 hours 5 minutes) would equate to a near 4 minute (3 minute and 41 seconds) increase in race time. It may be more difficult than we think to break the 2 hour barrier, despite the argument that a 2-hour marathon is possible with cooperative drafting, an ideal course and weather conditions and lighter, more compliant running shoes [53].

We specifically chose to perform the runs when the outdoor ambient temperature and relative humidity were relatively low in order to prevent any heat-associated increases in E_run_. Body temperature would rise primarily from metabolic heat generation, for which we estimate 90±10 kJ/kg based on our mean E_run_ values. Assuming 50% of this heat was lost by conduction/convection, the remaining heat would necessitate 1.1±0.3 L of sweat in order to lose heat by evaporation (for subjects ranging in mass from 45 to 73 kg). Our subjects may have had a reduction of body mass over the course of the run so their E_run_ should have been divided by a smaller body mass. Estimates of muscle energy cost and tendon energy return (J•stride^-1^) would also be affected by this possible change in body mass; a lower body mass would require a lower F_z_ at a given speed [35] as well as a lower tendon energy storage. If we assume a weight change of 1 kg following the run, then the post-E_run_ is 3.0% higher than the E_run_ measured prior to RUN. In order to try to confirm this hypothesized weight change, we later measured the weights of trained male (n = 18) and female (n = 7) runners (some of whom had also participated in the original investigation) following a 90 minute run of similar relative intensities and where ingestion of fluids including water was not permitted. The average weight loss following RUN in this group of runners (pre-RUN mass = 73±14 kg) was 0.9±0.7 kg (1.2±0.8% of pre-RUN body mass). Therefore, by not accounting for the post-RUN change in body mass, we compute that the post-E_run_ should be ~3% higher than the E_run_ measured prior to RUN rather than the 1.3% when we assume the runner’s body mass does not change over the course of the RUN. It is also possible that the apparent decrease in energy cost in a few of our runners relates to a slightly greater than average weight loss during RUN.

We also observed a decrease in AT stiffness following RUN which was larger than the technical error in AT stiffness we have previously reported [29]. The reduced AT stiffness was a result of a greater AT elongation at any given absolute force post-RUN without a reduction in MVC force. Our finding that MVC force was only reduced 3% is contrary to previous prolonged running studies which report a significant decline in MVC following prolonged running. These reductions include a 17% reduction following two hours of treadmill running at 75% $\dot{\text{V}}{\text{O}}_{2}\text{max}$ [54] and a 30% reduction in plantarflexor force after a run of 24 hours. However, others have reported no significant reduction in maximal plantarflexor force after 4-hours of running [55]. It is likely that two or more mechanisms prevented reduction in MVC force with our runners. First, contrary to previous reports which measured MVC force following runs of longer duration, our run was of a shorter duration and at a constrained submaximal intensity. The run may not have been of sufficient duration and/or intensity to elicit a significant reduction in MVC force. Furthermore, as these runners were highly-trained and RUN was typically performed weekly, as part of their regular training, they may have adapted to perform a run of this duration without central or peripheral fatigue.

We also observed a significant increase in d_L_ at all absolute forces following RUN. Given the same AT forces throughout the run, an increased d_L_ is indicative of dynamic creep of the tendon. We estimate, based on the duration of RUN and the average stride length at the RUN speed [56], approximately 7500 AT loading cycles during RUN, which is consistent with previous literature for a similar 21 km run [25]. This should be sufficient to elicit dynamic creep in the AT [12,57] and therefore a significant reduction in AT stiffness post-RUN. Previous studies which could not demonstrate a significant reduction in AT stiffness post-run may not have been of sufficient duration [58] or speed [59] to cause dynamic creep since a minimum threshold may need to be achieved in order to elicit any change in AT mechanical properties [26,60]. Based on previous studies on dynamic creep of the AT [12,57], the increased d_L_ should occur early on in the run and reach a steady-state after approximately 30–35 minutes [61]. It would appear that when the contractions are of a sufficient magnitude to cause dynamic creep, this occurs early, but when the contractions are only of marginal magnitude, it may require more loadings to impact the stiffness. This may help explain the apparently-contrary observations in AT stiffness following RUN between the current study and previously published work.

A greater d_L_ without a change in AT force would result in a greater storage of AT strain energy during ground contact. However, in order to compensate for the additional d_L_, and assuming similar kinematics pre and post-RUN, an increase in muscle fascicle shortening is necessary. This additional shortening would result in an elevated level of muscle activation [10], and come at a significant muscle energy cost, which we have estimated following a prolonged run for the first time. Using our previous estimates of cross-bridge kinetics and energetics during steady-state running [27], from the assumed TS force generated during the stride and assuming no RUN-induced changes in kinematics, we show that the estimated change in d_L_ resulting from dynamic creep of the magnitude observed here would affect both the AT energy storage/release and the estimated TS muscle energy cost as a result of additional muscle shortening. Using a within-subject design here, we show a decrease in AT stiffness (9%) is associated with a 1.9% increase in muscle energy cost. The latter comes without an increase in AT strain energy storage and release.

Following RUN, the additional d_L_ resulted in a significant increase in the estimated muscle energy cost of 11 J•stride^-1^, without an increase in AT strain energy release. Therefore, we conclude that the storage and release of tendon strain energy by the AT is relatively less important for reducing whole-body E_run_. Rather, the limited lengthening of the AT during running serves to minimize muscle fascicle shortening and reduce the muscle energy cost [10,62,63]. In a previous study [47], we demonstrated that the magnitude of muscle fascicle shortening during the stance phase is similar to the shortening measured during running using ultrasonography [9]. This theoretical consideration, however, is difficult to show in vivo during running. Despite an increase in AT stiffness and reduction in E_run_, Albracht and Arampatzis [17] were unable to show any differences in muscle fascicle shortening during running, which may have been attributable to the difficulty in quantifying muscle fascicle shortening during running with ultrasonography and the sensitivity of this measurement. To further support the relationship between AT stiffness and E_run_, we demonstrate here that the change in E_run_ is also associated with a change in the estimated muscle energy cost. This additional muscle energy cost is a result of the predicted increase in muscle fascicle shortening following RUN. This additional shortening is a consequence of the mechanical fatigue of the AT.

We cannot disregard the substantial, albeit reasonable, assumptions made in estimating both AT strain energy return and muscle energy cost. These assumptions include, among others, constant ankle/knee joint kinetics prior to and following RUN. Furthermore, we ignore the energy cost of ion pumping associated with activation of the muscles. Differences in the estimated muscle energy cost we show post-RUN are a result of the estimated additional muscle shortening since we assumed no change in kinetics post-RUN, and thus the number of crossbridges required to support the force would be similar pre vs. post-RUN. Our conclusions regarding energy storage and return and muscle energetics must be tempered by the realization that actual measurements of tendon length change during running are challenging and do not always fit the theoretical expectations (Matijevich et al., 2018). Future research will aim to substantiate these assumptions by measuring running kinematics combined with muscle fascicle length change during the assessment of E_run_.

## Conclusions

In conclusion, the current results demonstrate dynamic creep of the AT during a prolonged run below the lactate threshold in trained male and female distance runners. The change in AT mechanical properties following RUN appears to have a small but significant effect on E_run_, presumable due to increased muscle energy cost. Calculations suggest that this effect is greater than the additional tendon strain energy release from the AT. These results further support the notion that a mechanically-optimal AT minimizes E_run_ in trained distance runners by minimizing muscle fascicle shortening, thus reducing muscle energy cost. Storage and release of strain energy by the AT is not an important contributor to minimizing E_run_.

## Supporting information

S1 File Supplementary data. (XLSX)

## Abbreviations

ANOVA: analysis of variance
AT: Achilles tendon
d_L_: tendon elongation
E_run_: energy cost of running
F: force
F_z_: vertical ground reaction force
MG: medial gastrocnemius
MVC: maximal voluntary contraction
RUN: prolonged 90 minute run
SD: standard deviation
sLT: speed of lactate threshold
SWC: smallest worthwhile change
TS: triceps surae
$\dot{\text{V}}{\text{O}}_{2}\text{max}$: maximal oxygen uptake
